# Highlights of International Conference on Medical Physics (ICMP- 2008)

**Published:** 2009

**Authors:** S. D. Sharma

**Affiliations:** Radiological Physics and Advisory Division, Bhabha Atomic Research Centre, CTCRS, Anushaktinagar, Mumbai – 400 094, India E-mail: sdsbarc@gmail.com

The International Conference on Medical Physics (ICMP-2008) and the 29^th^ Annual Conference of the Association of Medical Physicists of India (AMPI) was organized from 26 to 29 November, 2008, at the Multipurpose Hall of the Bhabha Atomic Research Centre (BARC), Anushaktinagar, Mumbai - 400094, India. The theme of the conference was “Advanced Technology of Radiation Medicine and Medical Physics Practice”. About 650 delegates from India and abroad, including 120 radiological / medical physics students from different universities of India participated in the conference. ICMP-2008 was inaugurated by Dr. Anil Kakodkar, Chairman, Atomic Energy Commission and Secretary to the Government of India, Department of Atomic Energy (DAE). While delivering the inaugural address, Dr. Anil Kakodkar summarized the importance of ionizing radiation in daily life, and the contributions of DAE / BARC toward the cancer control program of the country. He also stated that a sufficient number of trained technical manpower for safe medical applications of ionizing radiation is available in the country. However, there is a need to take the advanced technology equipment to the rural sector, improve the working conditions at rural medical institutions and remunerate the technically trained people adequately. In addition, he emphasised the need to restructure the training modules so that the trained manpower could efficiently use the advanced technology equipment for its intended applications. Shri S. K. Sharma, Chairman, Atomic Energy Regulatory Board (AERB) presided over the inaugural function and released the Souvenir and Book of Abstracts. In the presidential address, Shri S. K. Sharma highlighted the role of AERB in ensuring the safety and security of radiation sources. He also stated that though the radiation safety record in medical applications of ionizing radiation is very good we must be cautious while using advanced technology equipment for radiodiagnosis and treatment. Prof. Bhudatt Paliwal, Director of Medical Physics, Department of Human Oncology and Medical Physics, University of Wisconsin, Madison, USA, presented the keynote address where he described in detail the dosimetry problems associated with organ movements during intensity modulated radiotherapy (IMRT). The keynote address highlighted the feasibility of real-time motion tracking methodology during the delivery of IMRT. Dr. Anil Kakodkar felicitated five Ex-BARC scientists for their contribution to AMPI (See Annexure-1 for their brief bio-data).

One hundred eighty five papers, including the AMPI Ramaiah Naidu Memorial Oration, 29 invited, 35 oral and 120 poster papers were presented during the conference. Shri P. S. Viswantahan, Former Head, Department of Medical Physics, Tata Memorial Hospital, Mumbai, delivered the seventeenth AMPI Ramaiah Naidu Memorial Oration (RNMO). The RNMO award is bestowed on an eminent personality who has a long working experience in the field of medical physics with a good track record of academics, research, and clinical practice. The title of his deliberation was “Medical Physics in India: History, Development, and Activities”. During his talk he described in detail the early days (1943) and the current medical physics activities in the country, including commercially available technology and indigenous developments. He also listed the future directions to deal with hi-tech equipment and emphasized the need for harmonization in medical physics training modules. The key feature of ICMP-2008 was the AMPI-AROI (Association of Radiation Oncologists of India) joint scientific meeting on November 28, 2008, at Nehru Centre, Worli, Mumbai. The joint meeting was the first of its kind, which could be assumed to be the defining moment in the history of medical applications of ionizing radiation in India. Both the clinical and physical aspects of recent radiation oncology, such as, IMRT, IGRT, precision radiotherapy by Cyber Knife, adapted Telecobalt machines and Proton accelerators, Image guided brachytherapy, and indigenous development of radiotherapy technology were presented during the joint meeting. The joint meeting was useful for radiation oncologists, for improving their understanding of medical physics and the technological aspects of recent radiotherapy, while it was equally beneficial for medical physicists to understand the clinical requirements and associated complexities when implementing hi-tech radiotherapy. Panel discussion on “Newer Technologies: Promises and Pitfalls” witnessed the active participation of a large number of medical physicists and radiation oncologists. It was concluded during the discussion that technology is available for high precision radiotherapy and dose escalation, but it should be used discriminately as a majority of cancers require palliative treatment by conventional techniques.

The scientific deliberations of ICMP-2008 covered the whole spectrum of medical radiation physics: Radiation Therapy Physics and Devices; Medical Imaging Physics and Devices; Radiation Dosimetry and Standards; Radiation Physics; Radiation Biology; Time Dose Models; Commissioning, Quality Assurance, and Audits; Clinical Aspects of Radiation Oncology; Clinical Aspects of Medical Imaging; Computational Tools in Medical Physics; Education and Training in Medical Physics; and Radiation Protection and Safety. Presentations on recent developments in the technology of radiation medicine and methodology of imaging and radiation therapy were the special attractions. The oral and poster presentations were evaluated for the best Oral and Poster articles. At the end of the conference “Improvement of ImatriXX in terms of spatial resolution and large field acquisition for patient specific IMRT verification” by Arun S. Oinam, Lakhwant Singh, Pradeep Goswami, Anup Bhardwaj, B. S. Rana, S. C. Sharma, from the Radiotherapy Department, Postgraduate Institute of Medical Education and Research, Chandigarh, India, and “Dosimetry of in-house designed circular cone for stereotactic treatment using MVCBCT” by Kamlesh Kumar Gupta, V. K. Sathiyanarayanan, Janhavi R. Bhangle, R. Vaitheeswaran, from the Department of Radiation Oncology, Ruby Hall Clinic, Pune, India were declared the best Oral and Poster papers respectively.

The overwhelming participation of trades dealing with medical radiation equipment, dosimetry systems, phantoms, computerized treatment planning systems, and treatment accessories was the other attraction of ICMP-2008. A number of recent technological equipments (Cyber Knife, 4-D medical linear accelerator, Gamma Knife Perfexion, Image Guided HDR Brachytherapy Systems) and dosimetry systems (2-D array with high resolution), phantoms (Dynamic phantoms to simulate organ movements), patient immobilization devices (SBRT immobilization systems), and other related products were demonstrated in 32 stalls arranged near the conference venue.

In summary, the conference deliberations were useful for radiation scientists, medical physicists, radiation oncologists, radiologists, radiobiologists, dosimetrists, and radiation technologists.

## Annexure-1: Brief bio-data of senior AMPI members and ex-BARC scientists who were felicitated during ICMP-2008

**Dr. B.C. Bhatt**

Dr. B. C. Bhatt served AMPI in various capacities. He was Secretary, AMPI Executive committee during 1995-’97; President, AMPI Executive committee during 2003-’05; and Chairman, AMPI Board of Trustees during 2005-’07. As a university teacher, Dr. Bhatt guided a number of M. Sc. and PhD Students in the field of radiation dosimetry and medical physics. He was Chairman, AERB Over-Exposure Committee, from 1997 to 2000; Chairman, AERB Task Group for review of syllabi of various training courses; and Chief coordinator of the HBNI (Homi Bhabha National Institute) Committee to review the Dip. R. P. syllabus. Dr. B. Bhatt was the Head, RP AD, BARC, from 1997 to 2004, where he was closely associated with medical advisory services for the country. Presently, he is working as Emeritus Scientist of CSIR.

**Dr. A. S. Pradhan**

Dr. A S. Pradhan has been an active member of AMPI since its inception. He has worked as the convener of Scientific Program Committees of several AMPI conferences, including ICMP-2008. He was Secretary, AMPI Executive Committee (EC) from 1999 to 2001; President, AMPI EC from 2001 to 2003 and Chairman, AMPI Board of Trustees from 2003 to 2005. He played a pivotal role in arranging the waiver of the backlog annual membership fee of the International Organization of Medical Physics (IOMP) and ascertaining the continuity of affiliation of AMPI to IOMP. As a recipient of Alexander-von-Humboldt (AvH) Fellowship of Germany, he worked on the use of TLDs for low and high LET radiation therapy beams, which is widely acknowledged. He guided a number of medical physicists for the PhD degree. Since 2006, he is the editor-in Chief of Journal of Medical Physics. He also continues to be a member of the International Commission on Radiological Protection (ICRP) Committee-2 on “Doses from Radiation Exposure” and on the editorial board of the Radiation Protection Dosimetry journal of UK.

**Dr. O. P. Massand**

Dr. O. P. Massand has been associated with AMPI for over three decades. He was the convener AMPI Trust when various bye laws were formulated to establish the Trust. He served in this capacity for six years during which period the financial position of AMPI was consolidated. He was Vice-President of AMPI for four years, from 1995 to 1999, and was actively associated with the organization of three national and one international conference during this period. Presently he is Chairman, Board of Trustees. He developed CR-39, an SSNTD based system for Neutron Personal Monitoring, which is routinely being used since 1989. He served as an IAEA expert in Sri Lanka. He retired as Head, Personnel Monitoring Section, BARC, in March 2004.

**Shri U. B. Tripathi**

Shri U. B. Tripathi joined the AEET in the Hospital Physics Group of the Directorate of Radiation Protection (D.R.P) in December 1965. He had been continuously working in the medical physics group till the date of his superannuation on 30 November, 2004. He is a founder member of AMPI and has shouldered various responsibilities such as Chairman AMPI Trust; member AMPI EC, and Election Officer. He organized and conducted a number of AMPI workshops for the benefit of clinical medical physicists. He was associated with medical Physics activities in various capacities in general and with the Dip. R. P. program in particular. He was a demonstrator as well as lecturer of this course since 1966. He also served as Chairman, Board of Examiners of this course for more than a decade.

**Dr. A. Shanta**

Dr (Mrs.) A. Shanta rendered her services to AMPI in various capacities and has contributed significantly to the activities of the association. She is the Associate Editor of Journal of Medical Physics for more than a decade. Her contribution toward the development, quality assurance, and dosimetric aspect of any new brachytherapy technique / source, introduced in India, is commendable. It includes the introduction of the indigenously produced ^60^Co tubes and needles, replacement of radium with ^137^Cs sources, introduction of ^192^Ir wire sources for interstitial applications, introduction of high dose rate brachytherapy, indigenously produced ^125^I seeds for ophthalmic applications, and intracoronary brachytherapy. She worked as a professional for three years at the Dosimetry Section of the International Atomic Energy Agency (IAEA), Vienna. During this period, she initiated a project for implementation of international traceability for calibration of brachytherapy sources and contributed to two TECDOCs, published by the IAEA. Dr (Mrs.) A. Shanta retired from RP AD, BARC after nearly 40 years of valuable service.

